# Beyond “Brain Drain” Versus “Brain Circulation”: Networked Governance and the Agentic Internationally Qualified Nurses in Transnational Nursing

**DOI:** 10.1177/15271544251339675

**Published:** 2025-05-14

**Authors:** Animesh Ghimire

**Affiliations:** 1 School of Nursing and Midwifery, 2541 Monash University, Clayton, VIC, Australia

Dear Editor-in-Chief,

I write as a migrant nurse and nursing academic in Australia, inspired by the recent article on internationally qualified nurse (IQN) retention in New Zealand, “Nursing Brain Drain, How Do We Retain Our Internationally Qualified Nurses: A Close Examination of Push and Pull Factors” (Ram et al., 2025). I commend the authors for highlighting the factors that prompt IQNs to leave New Zealand, yet I believe there is room to expand these ideas by examining the multidirectional agency of migrant nurses, the urgent need for networked governance, and the ethical dimensions of our transnational labor market. My own journey—having migrated from one country to another—affords me firsthand insight into the complexities underlying these moves, which cannot be fully explained by the “brain drain” or “brain circulation” models alone.

## Asymmetrical Power Dynamics and Ethical Responsibility

While nurses from the top three source countries, India, the Philippines, and Fiji, seek better salaries and professional growth in countries such as New Zealand (11.7 nurses per 1,000 population) and Australia (13.7 per 1,000), their home countries grapple with nurse-to-population ratios far below the World Health Organization's (WHO) recommended threshold (WHO, 2022). India stands at merely 1.7 per 1,000, the Philippines at 4.8, and Fiji at 3.8 (WHO, 2022). The gulf between source and destination nations is stark, raising ethical concerns around equity and global health. High-income countries (HICs) benefit from recruiting well-trained nurses, yet they bear an obligation to mitigate any negative consequences of the outflow from already under-resourced systems.

## Beyond Bilateralism: The Need for Networked Governance

Existing agreements, such as the Trans-Tasman Mutual Recognition Act (TTMRA) (Nursing Council of New Zealand, 2024), streamline the movement of professionals between Australia and New Zealand but fail to address the larger regional nursing labor market. A promising path forward would be creating a multilateral workforce governance structure representing both source and destination countries. Such a council or consortium could coordinate:

1. *Ethical Recruitment Guidelines:* Reinforce the WHO Global Code of Practice in a binding or semibinding manner (WHO, 2024), ensuring that foreign nurse recruitment does not exacerbate shortages in source countries.
2. *Skills Transfer Mechanisms:* Formalize programs for IQNs to maintain connections to their home countries (e.g., short-term research exchanges and remote mentoring) to share their advanced expertise.
3. *Harmonized Workforce Planning:* Gather real-time data on nurse supply and demand across regions to avoid both understaffing in source countries and oversupply paradoxes in places like New Zealand.

## Reconceptualizing “Aid” as “Human Capital Investment”

Countries with high nurse demand often provide financial or technical aid to source nations, but these efforts are not always structured to build lasting capacities in the nursing workforce. A more integrated approach would systematically invest in nursing schools, leadership programs, and specialty education tracks explicitly linked to migration pathways (WHO, 2020). This strategy allows IQNs to contribute to their home country's healthcare infrastructure, even if their primary employment is abroad. Rather than merely funneling money into broad infrastructure projects, focusing on targeted human capital investment can create sustainable, mutually beneficial outcomes.

## The Agentic IQN

As a nurse who has personally navigated these transnational channels, I emphasize that IQNs are not passive participants pushed and pulled by rigid economic forces. In forging their own career trajectories, many develop advanced clinical, cultural, and leadership skills in high-income settings—skills that can be shared transnationally and adapted to fit varying health contexts. The end result can be a genuine “exchange” rather than a unidirectional “drain,” but only if policies and structures support such reciprocity.

## Implications for Nursing Practice

This multilayered process demands that nurse leaders recognize and integrate the perspectives of migrant nurses at every level of policymaking and practice. Whether establishing mentorship programs, designing continuing education initiatives, or advocating for inclusive workforce policies, a globally attuned nursing sector acknowledges that our profession is inextricably interlinked across borders.

IQN mobility can be an opportunity rather than a problem—if we shift from an isolated, bilateral lens to a more robust, networked governance system. By embracing ethical recruitment, strategic human capital investment, and meaningful skill exchange, we stand to elevate nursing globally, ensuring that the benefits of migration extend to all parties: source countries, destination nations, and, most importantly, the nurses who traverse these divides with hope, expertise, and ambition.

Sincerely,

Animesh Ghimire, MPH, M. Ed, MSN, BSN
